# Clinical performance of stem cell therapy in patients with acute-on-chronic liver failure: a systematic review and meta-analysis

**DOI:** 10.1186/s12967-018-1464-0

**Published:** 2018-05-10

**Authors:** Ran Xue, Qinghua Meng, Jinling Dong, Juan Li, Qinwei Yao, Yueke Zhu, Hongwei Yu

**Affiliations:** Department of Critical Care Medicine of Liver Disease, Beijing You-An Hospital, Capital Medical University, No. 8, Xi tou tiao, You an men wai Street, Feng tai District, Beijing, 100069 People’s Republic of China

**Keywords:** Stem cell therapy, Acute-on-chronic liver failure, Clinical performance

## Abstract

**Background:**

Stem cell therapy has been applied in the treatment of acute-on-chronic liver failure (ACLF). However, its clinical efficiency is still debatable. The aim of this systematic review and meta-analysis is to evaluate the clinical efficiency of stem cell therapy in the treatment of ACLF.

**Methods:**

The Cochrane Library, OVID, EMBASE, and PUBMED were searched to December 2017. Both randomized and non-randomized studies, assessing stem cell therapy in patients with ACLF, were included. The outcome measures were total bilirubin (TBIL), alanine transaminase (ALT), international normalized ratio (INR), albumin (ALB), and the model for end-stage liver disease (MELD) score. The quality of evidence was assessed by GRADEpro.

**Results:**

Four randomized controlled trials and six non-randomized controlled trials were included. The TBIL levels significantly decreased at 1-, 3-, 12-month after the stem cell therapy (p = 0.0008; p = 0.04; p = 0.007). The ALT levels decreased significantly compared with the control group in the short-term (p < 0.00001). There was no obvious change in the INR level compared with the control groups (p = 0.64). The ALB levels increased markedly as compared with the control groups (p < 0.0001). The significant difference can be found in MELD score between stem cell therapy and control groups (p = 0.008). Further subgroup analysis for 3-month clinical performance according to the stem cell types have also been performed.

**Conclusion:**

This study suggests that the clinical outcomes of stem cell therapy were satisfied in patients with ACLF in the short-term. MSCs may be better than BM-MNCs in the stem cells transplantation of ACLF. However, more attention should focus on clinical trials in large-volume centers.

## Background

Acute-on-chronic liver failure (ACLF) is a global medical problem, with high prevalence and being serious, life-threatening in risk populations [1, 2]. ACLF occurs in patients with undiagnosed or previously diagnosed chronic liver disease (CLD) and was distinguished from acute hepatic damages such as coagulopathy and jaundice and complicated within 4 weeks because of sites and/or encephalopathy [3]. ACLF is usually accompanied by multiple organ failure, rapid progression and low survival rate. Liver transplantation is the sole solution that has proven beneficial, but the rapid disease progression and lack of donors limit its application [4]. Therefore, it is urgent to find a safe and effective therapeutic approach to ACLF.

Nowadays, a novel therapeutic strategy, stem cell therapy, is beginning to apply in the treatment of ACLF. Stem cells are a kind of undifferentiated cells, which capable of unlimited proliferation [5]. Cells such as hematopoietic stem cells (HSCs), mesenchymal stem cells (MSCs) and unsorted bone marrow cells (BMCs) have all been used in the stem cell therapy for liver diseases.

Several clinical researches have studied the consequences of stem cell therapy in patients with ACLF [6–8]. Ranged from early conceptual studies [9] to larger random controlled trials (RCTs), the results of those studies showed that stem cell therapy is safe and has beneficial effects on ACLF. However, these studies have several deficiencies such as the small size of the samples and the lacking of the control groups. Meanwhile, some studies have provided conflicting results about the effect of stem cell therapy [5, 10]. Therefore, it is still debatable for the effect of stem cell therapy in the treatment of ACLF. The purpose of this review is to evaluate the clinical efficiency of stem cell therapy in the treatment of patients with ACLF.

## Methods

This meta-analysis was adhered to the guidelines of Preferred Reporting Items for Systematic Reviews and Meta-Analyses (PRISMA).

### Literature search strategies and selection

We performed our data selection through four databases (PUBMED, EMBASE, Cochrane Library, and OVID) to Dec. 2017. The following medical subject heading (MeSH) terms were used: “hematopoietic stem cells”, “mesenchymal stromal cells”, “bone marrow”, “stem cells”, or “stem cell therapy”; “end stage liver disease”, “liver cirrhosis”, or “acute-on-chronic liver failure”. All literatures used in this study were published on humans and in English.

Two reviewers checked the abstract and title of publication independently. If the two reviewers identified the information relevant, we acquired the full text for a total review. Should any disagreements exist, we will discuss it with a third reviewer until there is a final conclusion.

### Data extraction and conversion

#### Data extraction

The picked articles were managed through EndNote X7 (Thomson Reuters, USA). Two independent investigators assessed the qualification of studies by the sequence of title, abstract, and full text. The following data were recorded from the articles: title, first author, journal, year of publication, characteristics of patients (i.e. age, sex), timing of detection (baseline, ongoing/post treatment), and accuracy of in vitro test (sensitivity and specificity).

#### Inclusion criteria and outcome assessment

The inclusion criteria were as bellows: (1) specific reporting the indications for stem cell therapy; (2) compared one of the following outcomes at least: alanine transaminase (ALT), total bilirubin (TBIL), albumin (ALB), international normalized ratio (INR), model for end-stage liver disease (MELD) score.

#### Exclusion criteria

The following trials were excluded: (1) it was difficult to extract or reasonably evaluate the data from the reliably articles; (2) those without clear outcomes; (3) abstracts, expert opinions, editorials, case reports, letters, and studies without control groups.

### Statistical analysis

The quality of evidence was assessed by GRADEpro [11, 12]. It is preferred that the data showed as changes. When changes were not available, but could be managed through the calculation of the data, they will be calculated through appropriate methods [13]. We performed the Meta-analysis by Review Manager (version 5.3). We use a random-effects model to evaluate the heterogeneity and I^2^ > 50% with p < 0.10 was statistically significant. The analysis of continuous variables was preceded by the mean difference (MD) as the summary statistic through the Inverse-Variance method, and the result was shown with a 95% confidential interval (CI). It was considered to be significant when p < 0.05.

## Results

### Literature search and characteristics

A total of 541 potentially eligible studies were identified and reviewed. 458 of records, of which titles or abstracts were screened, were excluded. 79 studies remained were evaluated in detail. And 69 of these studies were excluded, 7 of which with no comparative studies, 10 of which were reviews and meta-analysis, 23 of which were excluded for undesired treatment or unexpected inclusion of participants, 12 of which were involved patients not only ACLF, 15 of which did not report the outcome interested, and 2 of which were unpublished by English.

**Fig. 1:**
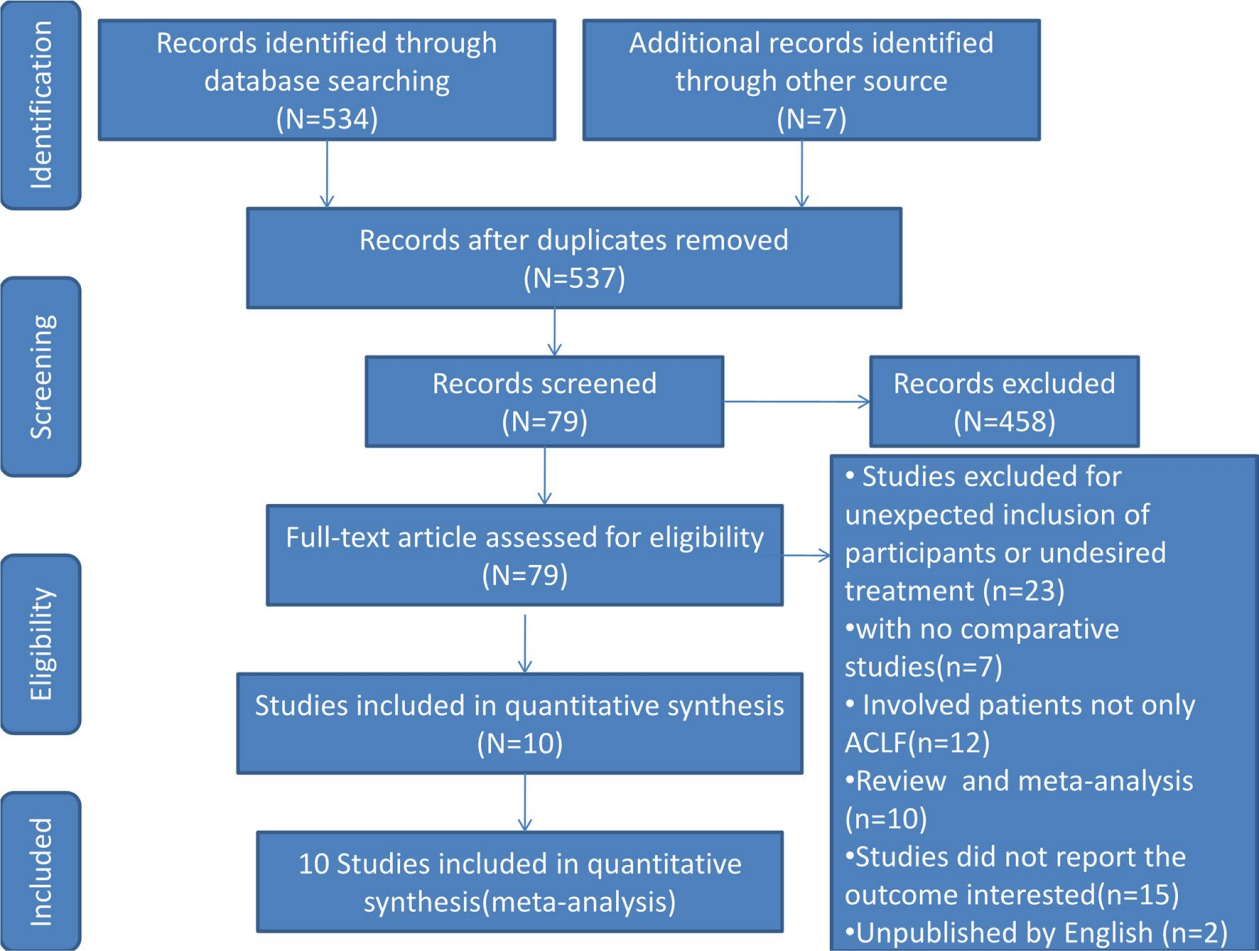
Flow diagram of the article selection process

As a result, 10 of the records were included in this meta-analysis, including five RCTs, five non-randomized controlled trial. A detailed flow chart of the study selection and the literature search is shown in Fig. 1. Although the authors were contacted, we were unable to obtain additional information to include in the analysis. The characteristics of the included studies are presented in Table 1.

**Table 1:** The characteristics of the involved studies

First authors/published year	Country	No.	Disease etiology	Study design	Cell source	Cell dosage	Injection rout	Sex (male/female)		Age (years)		End-point of observation	Quality
								SCT	SMT	SCT	SMT		
Ming Shi/2012	China	43	HBV-ACLF	RCT	UC-MSCs	Each 0.5 × 106/kg, thrice	PV	20/4	15/4	40 (24–59)	45 (26–62)	18 months	High
Bing-liang Lin/2017	China	110	HBV-ACLF	RCT	BM-MSCs	1.0 to 10 × 10^5^ cells/kg for 4 weeks	PV	53/3	51/3	42.8 ± 8.4	40.0 ± 9.9	24 weeks	High
Zheng Zhang/2012	China	45	HBV with decompensated LC	Open-labeled, paired, controlled study, CT	UC-MSCs	Each 0.5 × 10^6^/kg in NS, once every 4 weeks three times	PV	14/1	26/4	48 (25–64)	47 (29–64)	12 months	Low
Mehdi Mohamadnejad/2013	Iran	25	Decompensated LC (cirrhosis cryptogenic 11, PBC 2, HBV 2, HCV 1, AIH 9)	RCT	BM-MSCs	Median of 195 million (120–295 million) in 100 mL NS	PV	7/7	6/5	43.1 ± 17.6	34.6 ± 13.8	12 months	Moderate
Liang Peng/2011	China	158	LF caused by HBV	Retrospective case control study	BM-MSCs	In 10 mL of NS	HA	50/3	99/6	42.19 ± 10.8	42.22 ± 11.37	48 weeks	Very low
Yang-Qiu Bai/2014	China	47	Decompensated LC (43 HBV)	Prospective CT	BM-MNCs	1.0–11.2 × 10^10^/L	HA	20/12	9/6	46.4 ± 11.6	47.4 ± 11.1	24 months	Very low
Laurent Spahr/2013	Switzerland	58	Decompensated ALD (all with cirrhosis, 81% with alcoholic steatohepatitis	RCT	BM-MNCs	4.7 × 10^7^/kg	HA	24/4	20/10	54 (34–66)	56 (37–68)	3 months	Moderate
Qinzhi Deng/2015	China	70	HBV-related decompensated cirrhosis	Prospective study	APBSC	2–4 × 10^7^	HA	20/13	12/23	49.48 ± 11.07	50.20 ± 10.64	48 weeks	Low
Mehdi Mohamadnejad/2016	Iran	27	Decompensatedcirrhosis	RCT	BM-MNCs	20 mL of serum	PV	12/6	5/4	43.90 ± 14.84	46.22 ± 15.31	6 months	Moderate
Yu-Hua Li/2016	China	45	HBV-ACLF	Prospective study	UC-MSCs	100 × 10^6^ cells in 60 mL of NS	HA	8/3	26/8	51.1 ± 11.2	50.0 ± 10.9	24 months	Very low

*PBC* primary biliary cirrhosis, *HBV* hepatitis B virus, *HCV* hepatitis C virus, *AIH* autoimmune hepatitis, *BM-MNCs* bone marrow mononuclear cells, *HA* hepatic artery, *MSC* mesenchyme stem cell, *NA* not available, *RCT* randomized controlled trial, *PV* peripheral vein

### Stem cell therapy improved liver function and reduced liver injury

In advanced liver disease, the level of the serum ALB, which is a major protein produced by the liver, is reduced. INR reflects the coagulation function. The liver is the place where most of coagulation factors and variety of anti-thrombin synthesis were produced [2]. Advanced liver disease can significantly reduce the synthesis of coagulation factors, and affect the coagulation function.

**Fig. 2:**
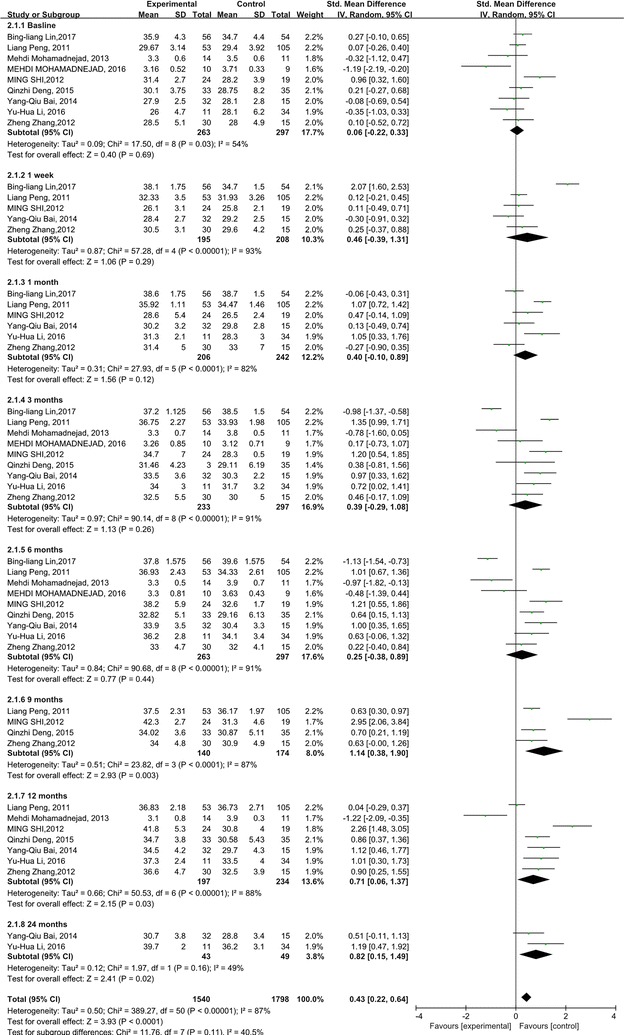
Forest plot of the ALB level during follow up

We analyzed the ALB and INR levels to investigate the impact of stem cell therapy on liver synthesis functions. Compared with the control groups, the ALB levels increased dramatically after the stem cell therapy (p < 0.0001). But heterogeneity should be considered about ALB level (p < 0.00001; with I^2^ = 87%). The subgroup analysis showed that the ALB levels increased significantly 9, 12, 24 months after the stem cell therapy (Fig. 2).

**Fig. 3:**
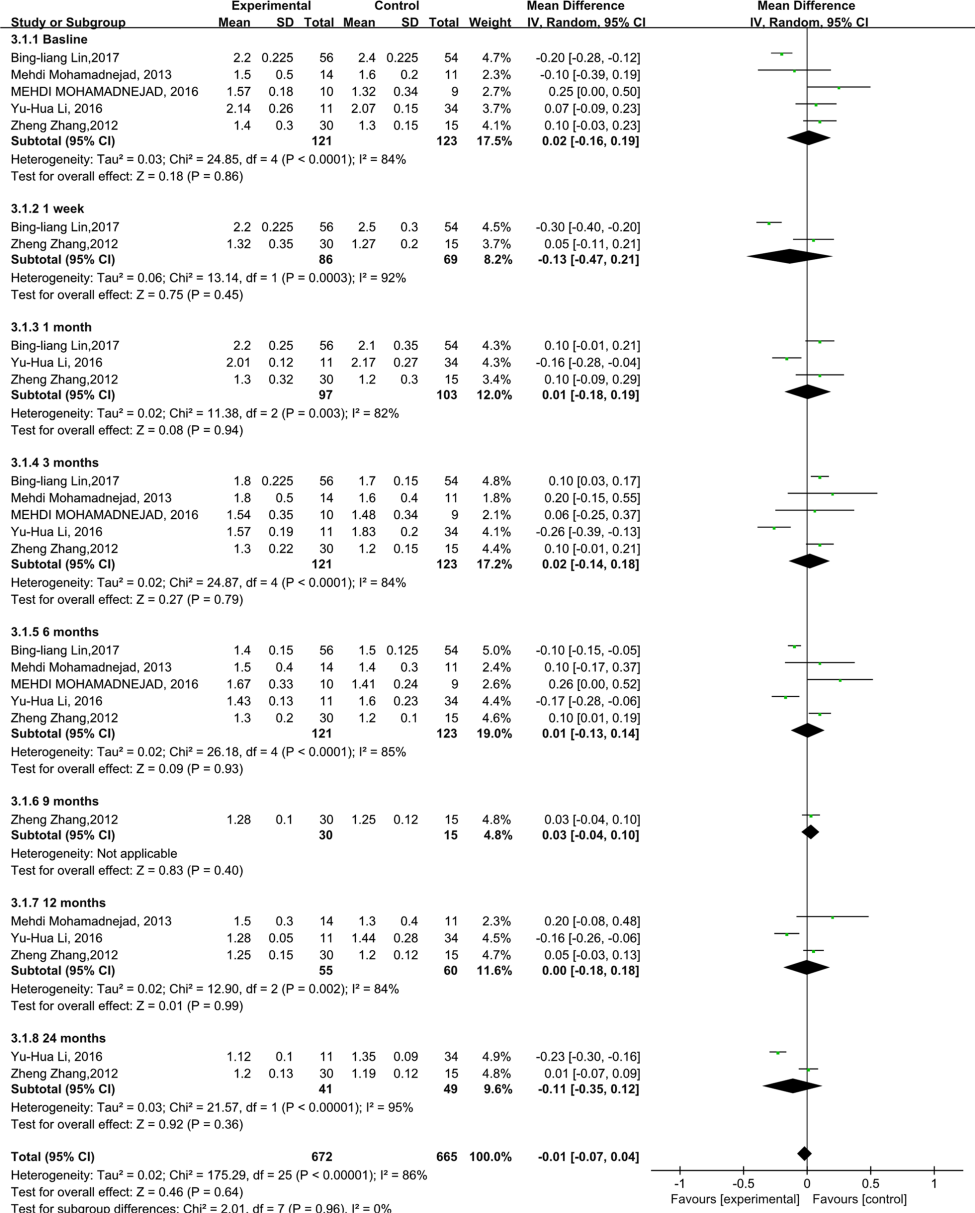
Forest plot of the INR level during follow up

The INR levels didn’t show any significant change compared with the control groups (p = 0.64). However, heterogeneity was especially high among these studies, although subgroup analysis was done (I^2^: 82–95%) (Fig. 3).

ALT is an enzyme in liver cells; it leaks into the general circulation when liver cells are damaged. It is considered to be a particular indicator of liver inflammation. The TBIL level can increase in biliary tract or liver diseases.

**Fig. 4:**
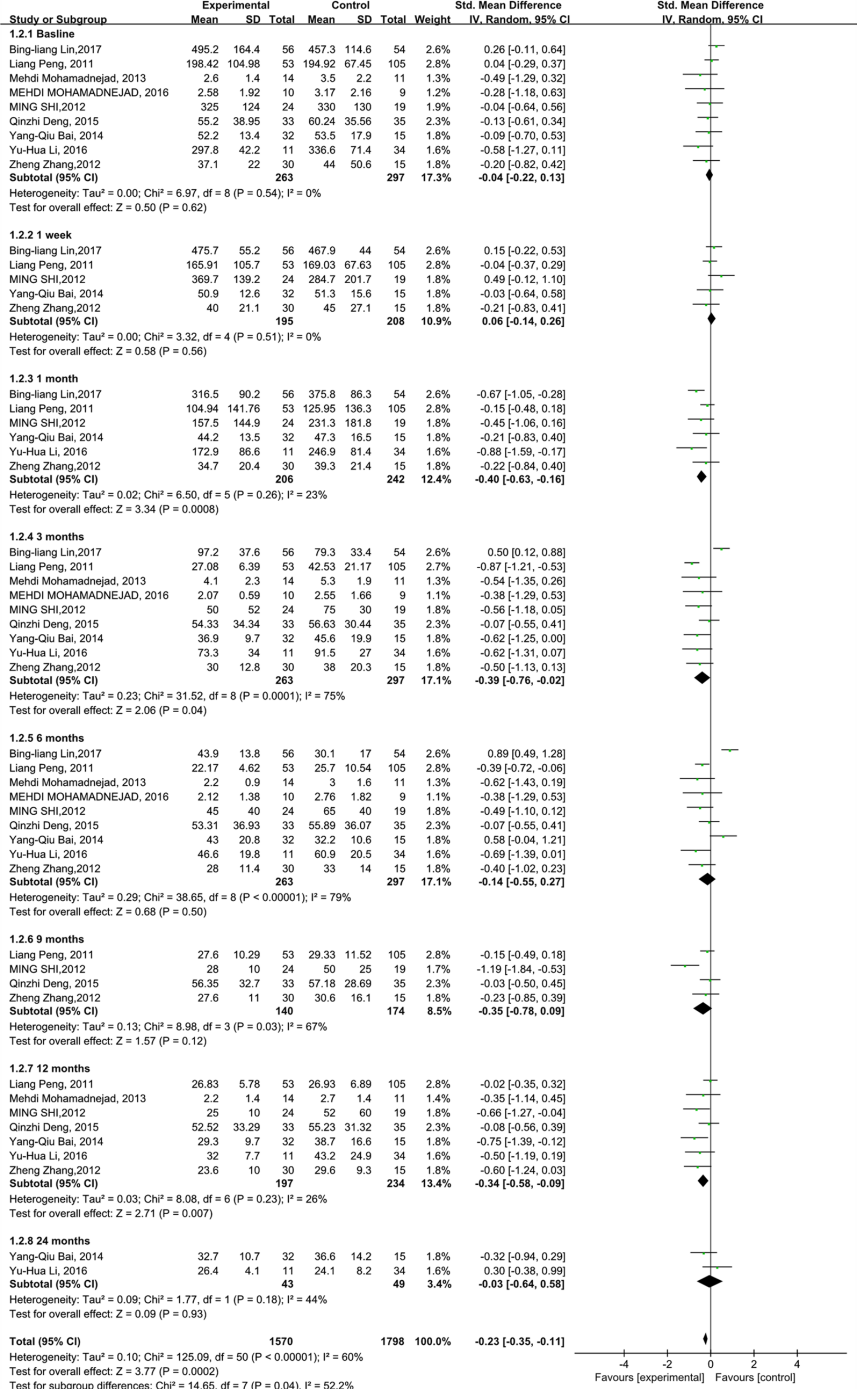
Forest plot of the TBIL level during follow up

In this research, the effects of the stem cell therapy on reducing liver damage were also evaluated through analyzing TBIL and ALT levels. The TBIL level significantly decreased 1, 3, 12 month after the stem cell therapy (p = 0.0008; p = 0.04; p = 0.007). Compared with the control group, no significant difference was found at 1 week, 6, 9 and 24 months time points (Fig. 4). No heterogeneity was detected regarding the TBIL level, except for 3, 6 and 9 months. But it could be explained by only one or two studies.

**Fig. 5:**
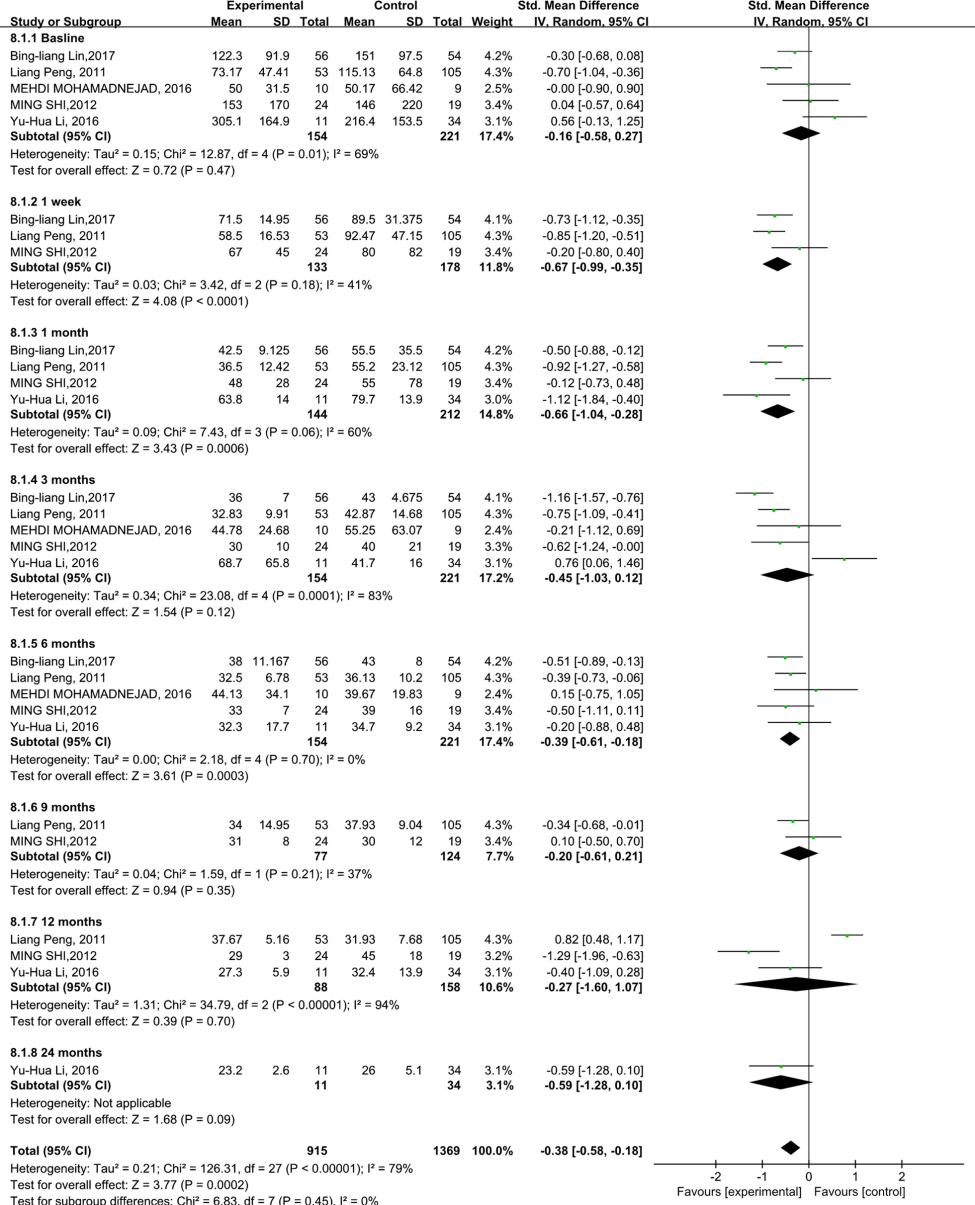
Forest plot of the ALT level during follow up

Compared with the control group, the ALT levels decreased significantly after stem cell therapy (p < 0.00001) (Fig. 5). The subgroup analysis showed that the ALT level in the stem cell therapy group also decreased, and was lower than baseline and the corresponding control group at the 1 week, 1 and 6 months time points. There were no significant differences in ALT level at 9, 12 and 24 months after stem cell therapy. No heterogeneity was found regarding the ALT level at 1 week, 6 and 9 months. However, heterogeneity should be taken into consideration for the ALT level at 1, 3 and 12 months. But it could be explained by only one or two studies.

### Stem cell therapy reduced the MELD score in ACLF patients

**Fig. 6:**
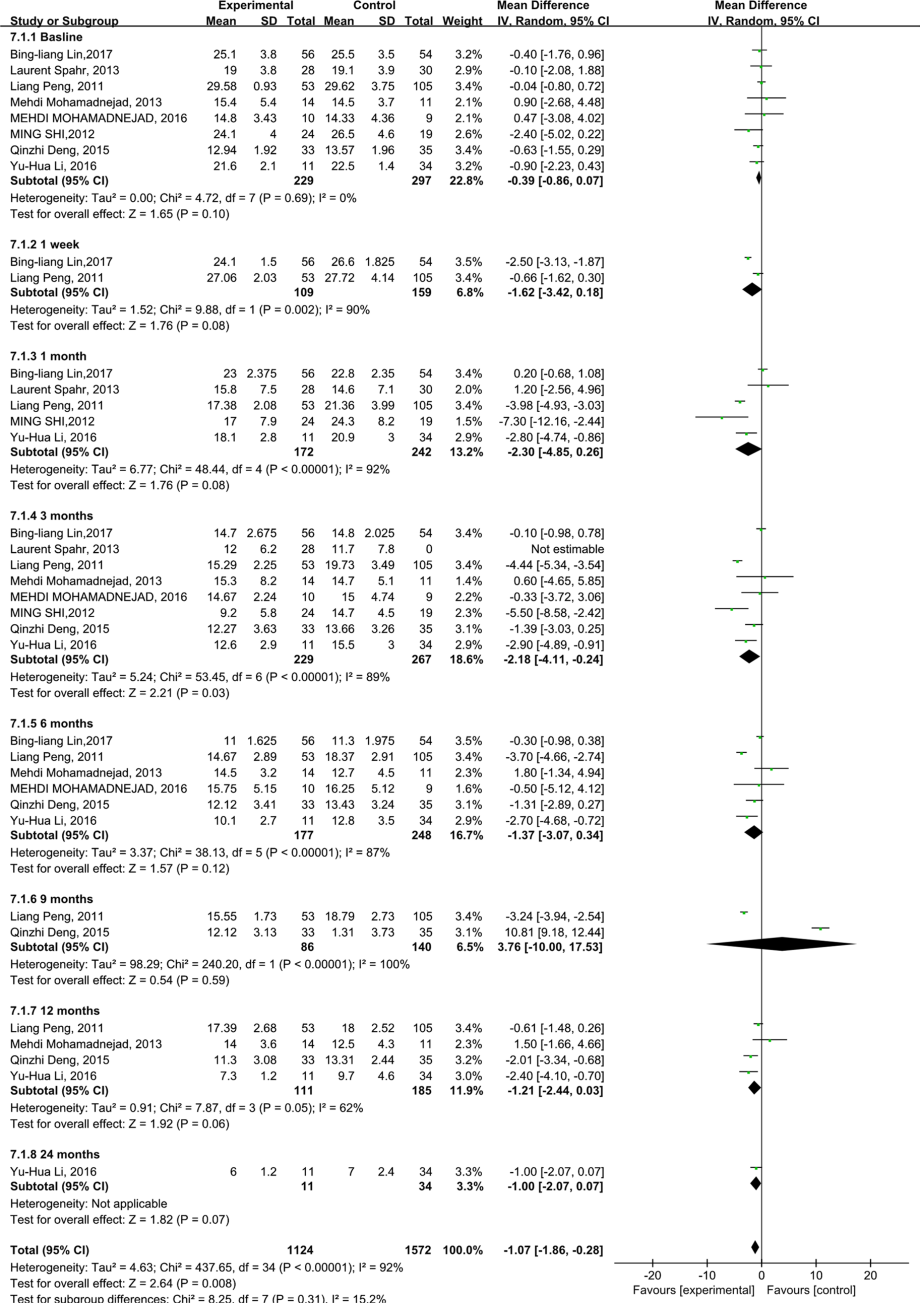
Forest plot of the MELD score during follow up

The MELD score is mostly used to assess the prognosis of end-stage liver diseases [14]. We observed that the MELD scores were both decreased in the stem cell therapy and control groups. Significant differences can be found between the stem cell therapy and control groups (p = 0.008) (Fig. 6). Moreover, heterogeneity was high within these studies, although subgroup analysis was done (I^2^:  62–100%). But it could be also explained by only one or two studies.

### Further subgroup analysis for 3-month clinical performance according to the stem cell types

**Fig. 7:**
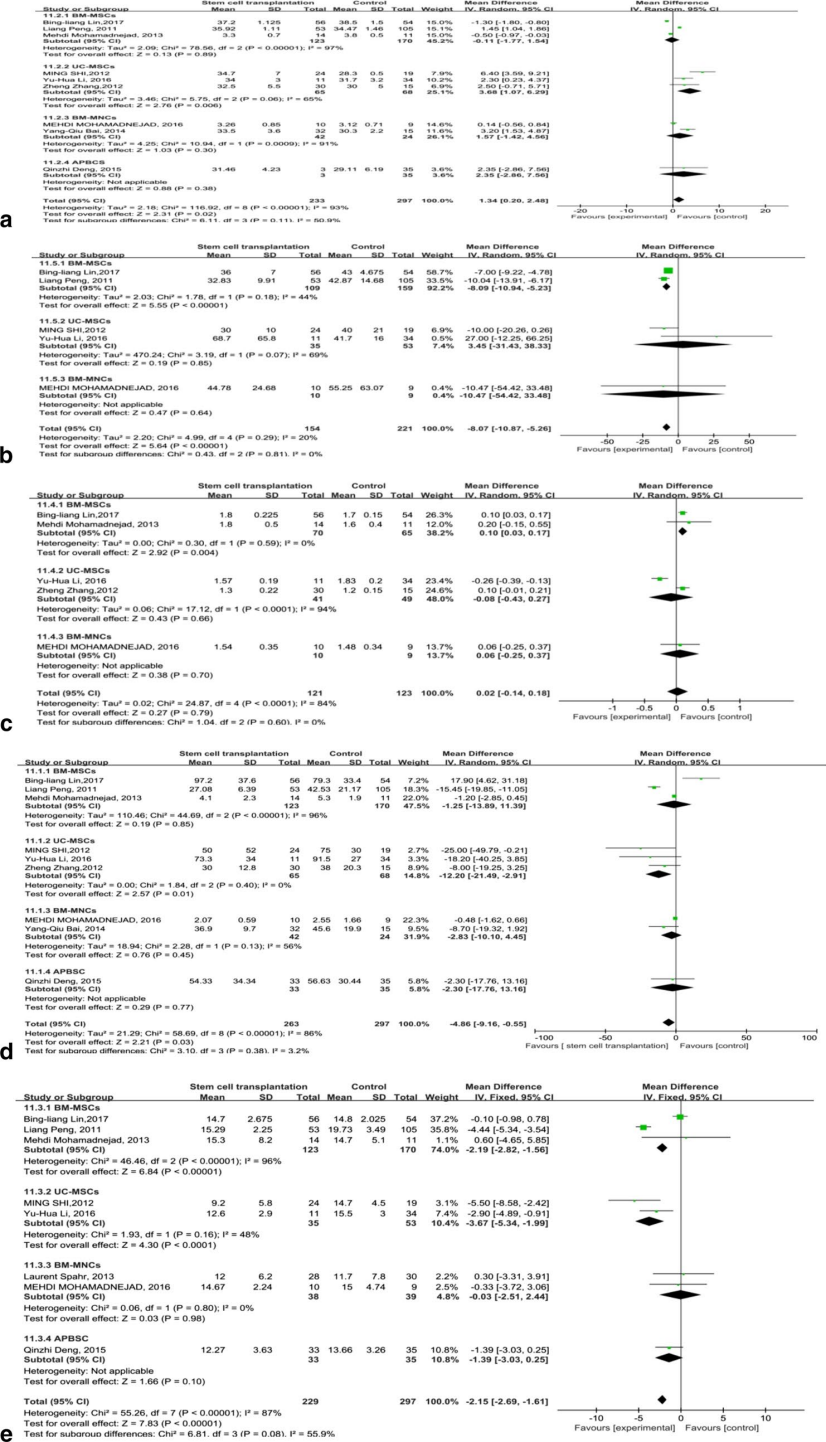
Futher subgroup analysis for 3-month clinical performance according to the stem cell types. **a** Forest plot of the ALB during 3 months. **b** Forest plot of the ALT during 3 months. **c** Forest plot of the INR during 3 months. **d** Forest plot of the TBIL during 3 months. **e** Forest plot of MELD scores during 3 months

Due to the high heterogeneity, we continued to perform subgroup analyses on the effects of stem cell type at 3 months after stem cell therapy (Fig. 7). For ALB, heterogeneity was still obvious in BM-MSCs and BM-MNCs (I^2^: 91–97%), not much high in UC-MSCs (I^2^ = 65%). It was shown that no heterogeneity was found for INR in BM-MSCs group (I^2^ = 0%). And INR had a significant change compared with the control groups (p = 0.004). However, it is the meta-analysis result of 2 studies. The credibility of the result is not high. As for TBIL, there were no differences for heterogeneity in UC-MSCs and BM-MNCs (I^2^ = 0%; I^2^ = 56%). It seems that UC-MSCs had a better effect on reducing the TBIL than BM-MNCs (p = 0.01). After the subgroup analysis on the effects of stem cell type at 3 months of follow-up for ALT, it was shown that no heterogeneity was found (I^2^ = 20%). Stem cell therapy can significantly reduce liver injury at 3 months of follow-up (p < 0.00001). BM-MSCs can obviously reduce the ALT level, which is better than other stem cell type (p < 0.00001). For MELD score, after subgroup analysis on the effects of stem cell type, there was still a high heterogeneity (I^2^= 87%), mainly in BM-MSCs group. It is indicated that UC-MSCs can significantly decrease the MELD score, which is better than BM-MNCs at 3 months of follow-up (p < 0.001).

### Methodological quality of the included studies

The quality assessment of each study is found in Table 1. The evaluation items were as follows: performance bias, detection bias, selection bias (random sequence generation, allocation concealment), attrition bias, reporting bias and other bias.

## Discussion

ACLF is a serious life-threatening disease with high prevalence. Due to the rapid progression and low survival rate of ACLF, it is urgent to find a safe and effective therapeutic approach to ACLF [1–3]. In this study, we found that stem cell therapy was able to improve liver function and alleviate liver damage. Meanwhile, stem cell therapy can also reduce the MELD score for ACLF patients. These results suggested that stem cell therapy can be considered to be a potential supplementary therapeutic approach to improve liver function in patients with ACLF.

The immunomodulatory and reparative functions of stem cells have been demonstrated to be therapeutically valuable for treating various diseases, including autoimmune diseases, diabetes, myocardial infarction, and organ therapy rejection [15–17]. The present systematic review and meta-analysis underlines the feasibility of stem cell treatment for patients with ACLF. In the process of transplantation, stem cells, especially MSCs, can not only differentiate into corresponding tissue cells, but also have strong immunoregulatory effects in various organs, such as inhibiting the proliferation of T cells in the recipient, altering the proportion of T cell subsets and the function of B cells, inhibiting dendritic cell differentiation and NK cell proliferation, reducing the number of inflammatory cytokines, increasing anti-inflammatory cytokines quantity, adjusting immune abnormalities and disorders, and improving the inflammatory environment around the damaged area [18, 19]. However, regardless of substantial clinical data, which proved the safety of stem cell therapy, there were no considerable supporting reports to confirm the immunological status of patients. As the immune profile of patients is the key to foresee the short and long-term graft clinical outcome, subsequent studies need to focus on monitoring of both clinical as well as immunological parameters [20]. Immunological parameters include T cells, regulatory T cells, B cells, NK cells, dendritic cell and intracellular cytokine expression.

As for the clinical monitoring, this study demonstrated that stem cell therapy improved the liver synthesis functions. ALB levels increased significantly 9, 12, 24 months after the stem cell therapy. But heterogeneity was still high among these studies during the 9, 12 months of follow-up, and could not be explained by only one or two studies. As for the heterogeneity of ALB level data, we considered that ALB transfusion may affect the serum ALB levels during the treatment of ACLF. Meanwhile, there existed heterogeneity at the baseline of ALB level in this study. In fact, ACLF is a dynamic process in which the variables at the time of hospitalization are predicted to vary over time, so the clinical processes and outcomes change accordingly [21–23]. Therefore, the change of ALB level between stem cell therapy and control group may be better for the assessment of liver function improvement than the assessment based on static baseline variables. However, there were not enough ΔALB data for this systematic review and meta-analysis now.

The INR levels did not show any obvious change compared with the control groups (p = 0.64). The reason may be that patients received plasma after admission. However, heterogeneity was high in these studies, although subgroup analysis was done (I^2^= 82–95%). More RCTs are urgently needed.

Stem cell therapy significantly decreased the TBIL levels at 1, 3, 12 months (p = 0.0008; p = 0.04; p = 0.007). The ALT level in the stem cell therapy group also gradually decreased and was lower than baseline and the corresponding controls at the 1 week, 1 month and 6 months time points. However, there were no significant differences in ALT level at 9, 12 and 24 months after stem cell therapy. Meanwhile, no significant difference was found in TBIL level compared with the control group at 9 and 24 months time points. It is indicated that stem cell therapy can alleviate liver damage in patients with ACLF in the short-term, but the long-term outcome for ACLF patients is not remarkable.

The MELD scores have been used to predict mortality of cirrhosis patients awaiting liver therapy, as well as prioritize liver allocation [24–27]. In this study, a significant difference can be found between the stem cell therapy and control groups (p = 0.008). It is suggested that stem cell therapy can reduce the MELD score and improve the prognosis of ACLF patients, so as to relieve the social burden for lack of liver donors.

There are some limitations in this study. We pooled different stem cell types in the publications, leading to a high heterogeneity degree. However, it was impossible to conduct all subgroup analyses according to the stem cell type. Because there were no enough data for us to do the subgroup analysis. Thus, we only selected 3 months for the subgroup analysis. It seems that the subgroup analysis can better reduce the heterogeneity in some degree. Compared with other types of stem cells, UC-MSCs had a better effect on increasing the ALB (p = 0.006). After the subgroup analyses on the effects of stem cell type at 3 months of follow-up for ALT and INR, BM-MSCs can obviously reduce the ALT level and INR, which is better than other stem cell types (p < 0.00001; p = 0.004). As for TBIL, it seems that UC-MSCs had a better effect on reducing the TBIL than BM-MNCs and BM-MSCs (p = 0.01). It is suggested that UC-MSCs and BM-MNCs can significantly decrease the MELD score, which is better than BM-MNCs at 3 months of follow-up (p < 0.00001; p < 0.0001). Above all, in our study, MSCs may be better than BM-MNCs in the stem cells transplantation of ACLF. However, more clinical trials with different stem cell types are needed to verify the clinical outcomes.

## Conclusions

This study suggests that the clinical outcomes of stem cell therapy were satisfactory in patients with ACLF in the short-term. Meanwhile, MSCs may be better than BM-MNCs in the stem cells transplantation of ACLF. Nevertheless, more attention should focus on large multicenter clinical trials and further basic research. Moreover, well-designed RCTs are needed to verify the clinical effectiveness of the long-term outcomes.

## Abbreviations

ACLF: acute-on-chronic liver failure
LT: liver transplantation
ALB: albumin
INR: international normalized ratio
MELD: model for end-stage liver disease score
ALT: alanine aminotransferase
TBIL: total bilirubin
MSCs: mesenchymal stem cells
HSCs: hematopoietic stem cells
BMCs: bone marrow cells
PRISMA: preferred reporting items for systematic reviews and meta-analyses
SMTs: standards medical treatments
MD: mean difference
CI: confidential interval
CLD: chronic liver disease
RCTs: random controlled trials
